# Procerenone: a Fatty Acid Triterpenoid from the Fruit Pericarp of *Omphalocarpum procerum* (Sapotaceae)

**Published:** 2014

**Authors:** Rosine Fotsing Ngamgwe, Raoul Yankam, Jean Rodolphe Chouna, Christian Lanz, Julien Furrer, Stefan Schürch, Marcel Kaiser, Bruno Ndjakou Lenta, Silvère Ngouela, Etienne Tsamo, Rudolf Brenneisen

**Affiliations:** a *Department of Organic Chemistry, Faculty of Science, TWAS Research Unit, University of Yaoundé 1, P. O. Box 812, Yaoundé, Cameroon.*; b *Department of Clinical Research, University of Bern, Murtenstrasse 35, CH-3010 Bern, Switzerland.*; c *Department of Chemistry, Faculty of Science, University of Dschang, P. O. Box 67, Dschang, Cameroon.*; d *Department of Chemistry and Biochemistry, University of Bern, Freiestrasse 3, CH-3012 Berne, Switzerland.*; e *Swiss Tropical and Public Health Institute, Socinstrasse 57, CH-4002 Basel, Switzerland. *; f *University of Basel, Petersplatz 1, CH-4003 Basel, Switzerland.*; g *Department of Chemistry, Higher Teacher Training College, University of Yaoundé 1, P. O. Box 47, Yaoundé, Cameroon.*

**Keywords:** Sapotaceae, *Omphalocarpum procerum*, Triterpenoid, Procerenone, Antiparasitic activity

## Abstract

Phytochemical investigation of a dichloromethane-methanol (1:1) extract of the fruit pericarp of *Omphalocarpum procerum* which exhibited antiplasmodial activity during preliminary screening led to the isolation of the new fatty ester triterpenoid 3β-hexadecanoyloxy-28-hydroxyolean-12-en-11-one (1), together with five known compounds 2-6. The structure of the new compound as well as those of the known compounds was established by means of spectroscopic methods and by comparison with previously reported data. Compounds 1- 4 were evaluated *in-vitro* for their cytotoxicity against L6 cell lines and antiprotozoal activities against *Plasmodium falciparum*, *Leishmania donovani*, *Trypanosoma brucei rhodesiense *and *Trypanosoma cruzi *(species responsible for human malaria, visceral leishmaniasis, African trypanosomiasis and Chagas disease, respectively). The tested compounds showed weak to moderate antiprotozoal activity and, no significant effect was detected regarding their cytotoxic potency.

## Introduction

*Omphalocarpum procerum *(Sapotaceae) is a tree confined to humid tropical Africa including Cameroon. It can grow up to 30 m in height and 40 cm in diameter. Its fruits are hard and often consumed by elephants (1, 2). In Africa, plants of the genus *Omphalocarpum *are prepared for various purposes such as decoctions, powders, macerations, and are used for years in traditional medicine to treat headaches; wounds skin diseases, constipation, elephantiasis, fever, cough, and rheumatism (3-6). Previous phytochemical investigations of plants of the Sapotaceae family have revealed the presence of alkaloids, phenolic compounds, glycerol, fatty acids, flavonoids, saponins and triterpenes (7-12). To the best of our knowledge, no phytochemical or pharmacological study has been reported on the species *O. procerum* so far. In a continuing search for bioactive compounds from Cameroonian medicinal plants, we have investigated the CH_2_Cl_2_-MeOH (1:1) extract of the pericarps of the fruits of *O. procerum. *Herein, we report on the isolation and structure elucidation of a new triterpenoid, procerenone (1), together with the antiparasitic activity of some isolated compounds.

## Experimental

*General procedure*

Melting points were determined on an M-540 melting-point unit (Buchi, Flawil, Switzerland). Optical rotations were measured, in chloroform solution, on a DIP-3600 digital polarimeter (JASCO, Tokyo, Japan). Infrared (IR) spectra were determined on a Fourier transform IR spectrometer (JascoP-2000). The mass spectra were acquired on a LTQ Orbitrap XL mass spectrometer (Thermo Fisher Scientific, Bremen, Germany) equipped with a nanoelectrospray ion source. Spectra were recorded in the positive ion mode with the resolution set to 100 000. Calibration of the instrument was performed with ProteoMass LTQ/FT-Hybrid ESI positive mode calibration mix solution (Supelco Analytical, Bellefonte, PA, USA). For MS/MS experiments, the precursor ion was isolated within a window of ±1.5 *m/z*-units. Collision-induced dissociation (CID) was performed using Helium as the collision gas and relative collision energy of 35%. The Xcalibur software package V. 2.0.7 was used for data processing. ^1^H and ^13^C NMR spectra were recorded on a Bruker Avance II 400 MHz spectrometer equipped with a 5-mm broadband probe head (BBI), operating at 400 (^1^H) and 100 MHz (^13^C), respectively. All chemical shifts are reported as relative differences to the internal standard tetramethylsilane (TMS). Silica gels of 230- to 400-mesh and 70- to 230-mesh (Merck, Darmstadt, Germany) were used for column chromatography (CC), respectively, while aluminum sheets precoated with silica gel 60 F_254_ (Merck) were used for thin-layer chromatography (TLC), with various mixtures of petroleum ether, *n*-hexane, EtOAc, and acetone as mobile phases.

*Plant material*

The Shell seeds of *O. procerum* were collected at Ambam in the Southern province of Cameroon. The plants were identified at the National Herbarium of Cameroon, where a voucher specimen (N°11955SFR) was deposited.

*Extraction and isolation*

The air-dried and powdered pericarps of the fruits (1.5 Kg) of *O. procerum *were extracted in a CH_2_Cl_2_-MeOH (1:1) mixture (5.0 L) at room temperature within 2 days. The solvents were evaporated under reduced pressure to afford 60 g of crude extract. The resulting mixture was successively extracted with *n*-hexane and EtOAc at room temperature to yield 22 g and 13 g of *n*-hexane and EtOAc extracts, respectively. The *n*-hexane soluble fraction was subjected to CC over silica gel (230 – 400 mesh) and eluted with mixtures of *n*-hexane-EtOAc and *n*-hexane-CH_2_Cl_2 _of increasing polarities. A total of 121 fractions of 300 mL each were collected and combined on the basis of similar TLC profiles to yield 3 main fractions (F_1_-F_3_). Fraction F_1 _(8.2 g) was a complex oily mixture that was not further studied. Fraction F_2_ (6.4 g) was subjected to CC on silica gel (Merck, 70 – 230 mesh, Merck) and eluted with *n*-hexane- EtOAc (1:0 to 1:1) to afford procerenone (1) (19 mg), stigmasterol (5) (49 mg), and betulin (2) (27 mg). Fraction F_3_ (5.8 g) was also subjected to CC on silica gel (Merck, 70 – 230 mesh) and eluted with *n*-hexane-CH_2_Cl_2_ (1:4 to 0:1) to afford β-amyrin (3) (50 mg), β-sitosterol (6) (62 mg), and lupeol acetate (4) (5 mg).

*Procerenone *(1): *3β-hexadecanoyloxy-28-hydroxyolean-12-en-11-one*

White powder. – [α]_D_^20^ 41.5 (*c *= 0.1, CHCl_3_). – IR (KBr, cm^-1^). – ν_max_ = 3447. 3 (OH), 1651.0 (C = O) and 1698.2 (C = O) cm^-1^. – ^1^H NMR (CDCl_3_, 400 MHz) and ^13^C NMR (CDCl_3_, 100 MHz) spectroscopic data, see Table 1. – HRESIMS: [M+H]^+^ at *m/z = *695.5955 (calcd.* m/z = *695.5973 for C_46_H_79_O_4_).

*Bioassays*

The *in-vitro *cytotoxicity and antiprotozoal activities against the parasites *T. b. rhodesiense*, *T. cruzi*, *L. donovani*, and *P. falciparum* were determined as earlier reported (20). The tests were carried out with the following strains, parasite forms and positive controls: *T. b. rhodesiense* STIB900, trypomastigote forms, melarsoprol, IC_50_ of 3 ng/mL; *T. cruzi*, Tulahuen C2C4, amastigote forms in L6 rat myoblasts, benznidazole, IC_50 _of 0.531 µg/mL; *L donovani*, MHOM/ET/67/L82, axenic amastigote forms, miltefosine, IC_50_ of 0.145 µg/mL; *P. falciparum*, NF54, erythrocytic stages, chloroquine, IC_50_ of 6 ng/mL and L6 cells, rat skeletal myoblasts, podophyllotoxin IC_50_ of 6 ng/mL.

## Results and Discussion

The air-dried and ground fruit pericarp of *O. procerum* was extracted at room temperature with a mixture of CH_2_Cl_2_–MeOH (1:1, v/v). The extract was concentrated to dryness under vacuum and the residue subjected to repeated column chromatographic separation to yield procerenone (1) along with betulin (2) (13), β-amyrin (3) (13), lupeol acetate (4) (14), stigmasterol 5 (15), and β-sistosterol (6) (16,17).

**Figure 1:**
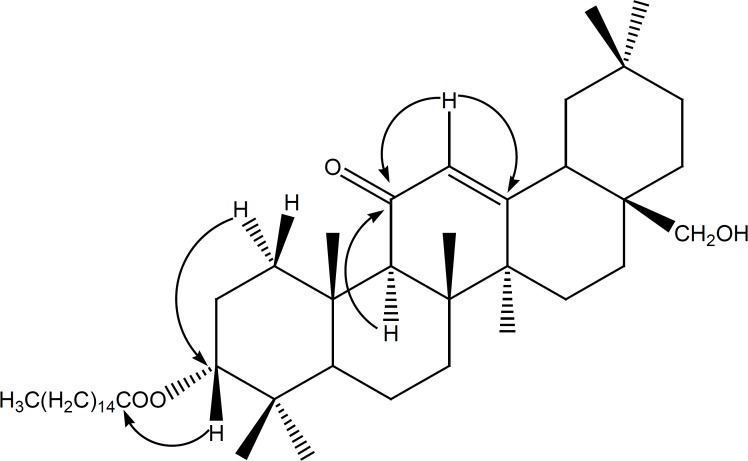
Selected HMBC correlations observed in compound 1.

**Figure 2:**
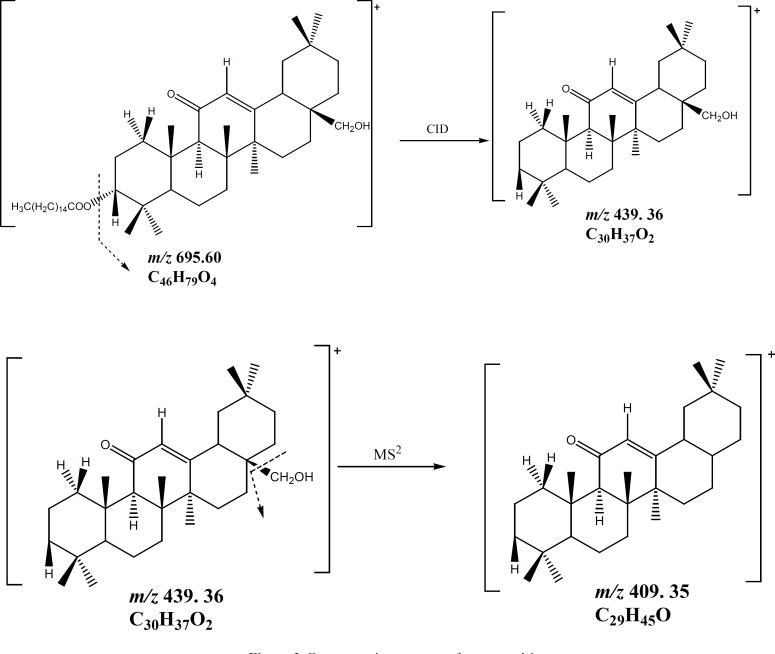
Fragmentation patterns of compound 1.

Compound (1) was obtained as a white powder. It gave a positive reaction to Liebermann-Burchard test, as usual for a triterpenoid. Its molecular formula C_46_H_78_O_4_, with eight double bond equivalents, was deduced from the HRESIMS spectrum which showed the pseudo-molecular ion peak [M+H]^+^ at *m/z *= 695.5955 (calcd.* m/z* = 695.5973 for C_46_H_79_O_4_). The IR spectrum showed characteristic absorption bands at 3447 (OH), 1651 and 1698 (α,β-unsaturated ketone), and 1613 (C = C) cm^-1^. The ^1^H NMR spectrum (Table 1) revealed the presence of seven singlet resonances due to seven angular triterpenoids methyl protons (δ_H _= 0.86, 0.90, 1.10, 1.14, 1.29, 1.39 and 1.59), a singlet of one methine proton (δ_H _= 2.37) and one singlet of an olefinic proton (δ_H _= 5.52). This spectrum also exhibited two doublets typical of an AB system (δ_H _= 3.45 and 3.20, *J* = 10.8 Hz) and one doublet of doublets (δ_H _= 4.47, *J* = 4.8, 12 Hz). Furthermore, the ^1^H NMR spectrum exhibited series of resonances at δ_H _= 0.88 (3H, t, *J* = 6.0 Hz), 1.26 (brs) and 2.27 (2H, t, *J* =7.5 Hz) which could be assigned to protons of a long alkyl chain. The ^13^C NMR (Table 1) and DEPT spectra of compound **1** displayed resonances characteristic of a single double bond (δ_C_ 128.2 and 169.6), one conjugated ketone carbonyl (δ_C_ = 199.7), one oxymethine (δ_C_ = 80.2), and one oxymethylene (δ_C_ = 69.5). The ^13^C NMR also confirmed the presence of the long chain acyl ester with the carbon resonances at δ_C_ = 173.5 (-COOR), 29.8 [(CH_2_)_n_] and 14.0 (CH_3_). The ^13^C NMR spectrum also displayed eight resonances typical for eight methyl groups, from which seven could be assigned to the triterpene pattern (δ_C_ = 16.3, 16.6, 18.6, 22.8, 23.2, 29.3 and 32.7) and one to a terminal methyl of the long acyl chain (δ_C_ = 14.0). On the basis of these NMR data, compound 1 was assumed to be a fatty acid ester of an olean-12-ene-type triterpenoid with one hydroxyl group and one α,β-unsaturated ketone group (12,18). The location of the ketone carbonyl at C-11 was deduced from the correlations observed in the HMBC spectrum between the olefinic proton H-12 (δ_H_ 5.52) and the carbons C-9 (δ_C_ 61.7), C-11 (δ_C_ 199.7) on one hand and between H-9 (δ_H_ 2.37) and the carbonyl carbon C-11 (δ_C_ 199.7) (Figure 1) on the other hand. According to these findings, the triterpenoid moiety of 1 was identified as being 11-oxoerythrodiol (2) (19). The ester function at the C-3 position was deduced from the correlation observed in the HMBC spectrum between H-3 (δ_H_ 4.47) and the ester carbonyl carbon (δ_C_ 173.5). The length of the acyl chain ester was deduced from the MS/MS^n^ spectrum which exhibits the characteristic ion peaks at *m/z* 677.58 [M–H_2_O]^+^, *m/z* = 665.59 [M–CH_2_O]^+^, and *m/z* = 439.36 [M–C_16_H_31_O_2_]^+^ (Figure 2). The axial orientation of the H-3 proton was deduced from the coupling constants with protons H-2 (δ_H3_= 4.47, dd, *J* = 4.4 and 12.0 Hz) (17). Thus, the structure of compound 1 was unambiguously assigned to 3β-hexadecanoyloxy-28-hydroxyolean-12-en-11-one, named procerenone.

**Figure 3:**
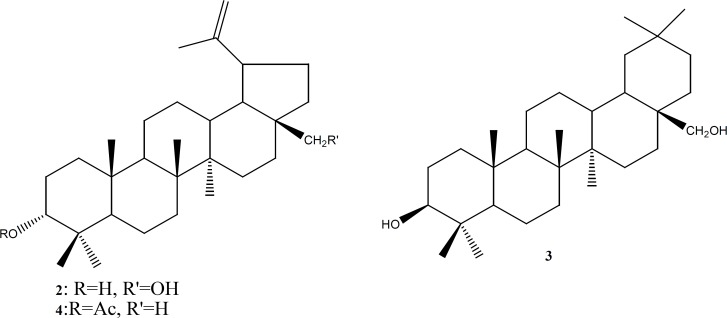
Structure of compounds 1**-**4.

Compounds 1-4 (Figure 3) were tested for cytotoxicity against L6 cell lines and for their antiprotozoal activity against *Plasmodium falciparum*, *Leishmania donovani*, *Trypanosoma brucei rhodesiense *and *Trypanosoma cruzi*. The tested compounds showed weak to moderate antiprotozoal activity against the tested parasites with IC_50_s in range of 9 to 80 µg/mL. No significant effect was detected regarding their cytotoxic potency.

## Conclusion

The phytochemical study of the fruit pericarp of *O. procerum* (Sapotaceae) led to the isolation and characterization of six compounds including one new fatty acid triterpenoids, procerenone. This class of secondary metabolite has been isolated from other genera of the Sapotaceae family like *Gambeya* and could be considered as one chemotaxonomic marker (12). The antiprotozoal activities of the tested compounds were nearly equal to that of the extract on each tested strain of parasite. Despite the moderate antiprotozoal potency of the extract it exhibited weak cytotoxicity to L-6 cell lines. The present results partially validate the use of *O. procerum* in folk medicine. 
